# Identification of Training Needs and Development of a Work-Integrated Training Program for Aged Care On-Site Pharmacists: Protocol for a Co-Design Study

**DOI:** 10.2196/95889

**Published:** 2026-07-31

**Authors:** Tiernan McDonough, Amy Page, Lisa Kalisch Ellett, Christopher Etherton-Beer, Aaron Davis, Ian Gwilt, Jacinta Johnson

**Affiliations:** 1 School of Pharmacy and Biomedical Science, College of Health, Adelaide University, Level 6, Bradley Building, Adelaide, SA, 5000, Australia, 61 0478602050; 2 Centre for Optimisation of Medicines, The University of Western Australia, Perth, Western Australia, Australia; 3 Western Australia Centre for Health and Ageing, University of Western Australia, Crawley, Perth, Australia; 4 Geriatric Medicine, Royal Perth Hospital, Perth, Australia; 5 Match Studio, Adelaide University, Adelaide, Australia

**Keywords:** lifelong learning, co-design, postgraduate education, aged care, pharmacist

## Abstract

**Background:**

From July 2024, Australian pharmacists are remunerated through federal funding to practice on-site within aged care homes. This Aged Care On-site Pharmacist (ACOP) program is novel in both Australian and international practice, aiming to improve the quality use of medicines for vulnerable older persons through medicines governance, person-centered medication reviews, and more. In this program, pharmacists are embedded in facilities and health care teams, varying from historical Australian models where they are visiting health care professionals. Distinct from pharmacist practice in hospital and community pharmacy, the role is professionally isolated from other pharmacists, limiting opportunities for peer collaboration and practice-specific professional development. Peer learning, mentorship, and workplace-based learning opportunities are considered integral for health professional development.

**Objective:**

This project aims to co-design a training program that provides a pathway to professional development for ACOPs aligned with the diverse interests of stakeholders, including health care professionals, residents, and informal caregivers.

**Methods:**

Focus group–style co-design workshops and stakeholder consultations will be used in this project. The “Double Diamond” framework will be used to structure the process, using 4 stages of alternating convergent and divergent exploration of stakeholder perspectives. A consumer advisory group has been engaged, consisting of persons with lived experience of aged care settings, for consistent observation and overarching guidance of the project. Exploratory workshops with pharmacists, allied health and medical professionals, residents, and families of residents will be conducted. Workshops will be audio- and video-recorded, transcribed verbatim, and analyzed using thematic analysis. Insights obtained from overarching themes as well as individual perspectives will be used to design a pilot program, which will be presented and discussed for feedback during follow-up focus groups. A pilot phase will then be undertaken with ACOPs, who will be further involved in feedback and workshop-style focus groups to collaboratively refine the program.

**Results:**

Funding for this project was granted through the Medicines Research Future Fund on March 27, 2023. Recruitment for preliminary phases began in January 2025, and data collection began in March 2025. Data collection is expected to end in October 2026. Ethics approval for this study was granted on December 19, 2024, by the University of South Australia Human Research Ethics Committee. Recruitment has exceeded the initial estimates of minimum participant involvement. Output data are expected to be available in early 2027.

**Conclusions:**

This will be the first co-designed workplace-based training program available for pharmacists undertaking the novel and complex role of ACOPs. It is expected to benefit the development of clinical and nonclinical skills, facilitate integration into existing teams, and offer a pathway to professional recognition.

## Introduction

### Pharmacists in Aged Care

As the international population ages and efforts continue to enable people to remain at home longer, the frailty and medical complexity of older adults entering aged care is increasing [1-4]. While many clinical and research efforts attempt to improve care for these people, medication-related harms persist internationally [5-7]. In the Australian context, this was most unambiguously identified with the Royal Commission into Aged Care Quality and Safety [8-13]. In an attempt to reduce these harms and improve care, the Aged Care On-site Pharmacist (ACOP) program was introduced in July 2024 in Australia [13]. This intervention provides funding for all residential aged care homes (RACHs) to integrate a pharmacist into the home to provide medicines expertise to existing teams. The broad indicative role descriptions have been provided, identifying an array of key tasks and responsibilities that ACOPs should undertake, including medicines reconciliation, comprehensive medication reviews, staff education, and more [14]. These role descriptions remain generalized and describe only what pharmacists may undertake, depending on the needs of residents and RACHs.

These are novel roles for pharmacists in Australia. Furthermore, they will be practicing in settings with limited professional contact with other pharmacists, and they will be operating within complex health care systems. Residential aged care has long been understood as a complex adaptive system marked by complex care needs, dynamic professional and social relationships, workforce issues, and more [15]. Early work on this role suggests the need for research on pharmacist education requirements in this role [16]. Dedicated training for pharmacists working in aged care settings both in Australia and internationally has been identified as integral to refine clinical and nonclinical skills, integrate into existing teams, and provide a pathway to recognition of expertise [17-22]. Preliminary Australian work has identified broad needs of pharmacist stakeholders, although this work was limited in its data collection methods (a single, brief workshop) and analysis (not described) [18].

Workplace-based training programs are available to other pharmacists practicing in specialist and complex roles, particularly those in hospital settings in Australia [23-25]. As health care, and specifically aged care, is considered a complex adaptive system, it is essential to engage stakeholders when implementing novel interventions that affect them [15,26,27]. Pharmacists working in such novel roles may further need to adapt to new ways of working given this new environment. Authentic end user engagement is essential to ensure that programs are fit for purpose. This project aims to leverage end user collaboration with researchers to identify the professional development needs of ACOPs, and subsequently develop a longitudinal, workplace-based training program that addresses these needs.

### Consumer Engagement

Consumer engagement, an authentic partnership with the end users and other stakeholders of an intervention or product, is considered essential for high-quality health care research [28,29]. For the purposes of this study, “end users” refers to pharmacists working in on-site aged care roles. “Other stakeholders” refers to the wider community of those impacted by interventions with the end users, such as health care staff, residents, and residents’ families. Meaningfully and collaboratively engaging stakeholders across all stages of research has a range of benefits. It aligns with bioethical principles, promotes creativity, fosters trust in health care, and represents an evidence-based method for improving research outputs [30-35]. The level of collaboration with nonresearchers exists upon a spectrum, from consultation and providing end users with information regarding the research, to the close collaboration and empowerment of end users to actively guide end products of research [36]. This is an important approach in the care of older persons, who are frequently excluded from health care research not only as participants in the research itself but also as partners in guiding research that affects them [37-39].

Co-design is a form of collaborative public participation that lies at the far end of the end user engagement spectrum. It is a creative and cognitive partnership between end users, other stakeholders, and researchers to better comprehend and accommodate the needs and circumstances of these end users [40]. In co-design methodologies, authentic partnerships between researchers and individuals who use or are affected by research outputs span all stages of the research process to plan, implement, review, and/or deliver research outputs. While researchers bring clinical or research expertise, individuals bring their lived experience expertise, providing a shared and balanced input into the final product [29,41,42]. A co-design methodology allows for integration and accommodation of these different perspectives in both the process and delivery of a new service such as that identified here.

An effective training and development program for pharmacists working in aged care should therefore be developed by drawing upon the insights of a range of stakeholders to ensure that it is fit for purpose and sustainable. Ultimately, it is believed that this co-designed program will assist in developing and building upon existing pharmacist skill sets to enact tangible, lasting, and evidence-based person-centered care and quality use of medicines.

### Objectives

The primary objective of this project is to develop a workplace-based training and development program for ACOPs that suits the needs and interests of the diverse stakeholders, including pharmacists, staff of aged care, and residents and their families.

## Methods

### Overview

Co-design methodologies will be used throughout this project to leverage the insights of end users and other stakeholders to collaboratively design a project that aligns with their diverse needs.

Co-design processes can exist across the participatory spectrum, with both contemporary and foundational participatory design literature highlighting that focusing only on decision-making power can be problematic and may miss opportunities for insights that can come from deep engagement processes with or without decision-making power [43,44]. This project will therefore focus on the co-design process as a collaboration between researchers, end users, and other stakeholders. Given the novelty of the ACOP role itself and the inherent uncertainty around its implementation, the co-design process will be focused on enabling contributions and exploratory insights rather than establishing a decision-making forum. Thus, although the researchers retain the relative majority of the decision-making power, we do not believe that this attenuates the authentic involvement of end users and other stakeholders in the processes we will undertake.

### Participants and Recruitment

A multilevel stakeholder design approach will be undertaken to facilitate the inclusion of not only end users and pharmacists but also the stakeholders who may be indirectly impacted by the intervention [45]. This enables widespread input that can increase the relevance and likelihood of implementation success [29,45]. Participants will be purposively sourced to obtain comprehensive perspectives of the diverse range of stakeholders of the intended training program. TM will make selection decisions after consultation with the wider research team. Maximum variation sampling will be used to explore a wide range of perspectives, appropriate given the objective of this project to create a training program suitable to a general cohort of aged care pharmacists. Although the pharmacists will be largely homogenous in their current role, variability in geographic settings, rurality, professional background, sex, and age will be sought. Pharmacists are eligible for inclusion if they have experience or interest in working within an aged care home as an ACOP. Pharmacists are eligible through both design and piloting phases regardless of whether they have completed Australian Pharmacy Council–accredited training as an ACOP (to explore existing gaps in initial programs) or they are qualified to work as an ACOP through historical qualifications. Pharmacists in the design phase will be eligible to participate in the piloting phase. Residents are eligible if they currently reside in aged care permanently, and families are eligible if they have had a family member reside in aged care permanently (either currently or in the past). Health care staff are eligible if they currently work within an aged care home (with or without an aged care pharmacist present). Minimum estimates of participant numbers that we aim to recruit are outlined below, stratified into groups involved in separate workshops. These estimates are driven by co-design methodological standards and feasibility of the project rather than “saturation,” a quantitative measure that may not fit the qualitative epistemological underpinnings of co-design. Information power rather than quantitative measures is prioritized in co-design methodologies [46].

The minimum target sample sizes that we aim to recruit are as follows:

- *Group 1 (pharmacists):* 8 pharmacists in the design phases and 8 pharmacists in the pilot and evaluation phases

- *Group 2 (residents and families):* 4 residents of aged care facilities and 4 family members of residents of aged care facilities

- *Group 3 (health care staff):* 1 registered nurse of an aged care home, 1 personal carer within an aged care home, 1 manager of an aged care home, 1 general practitioner working in an aged care home, and 2 allied health professionals working in an aged care home.

Pharmacists, health care staff, residents, and residents’ families will be recruited through advertisements, email invitation through professional networks of the investigators, social media advertising, and professional organizations such as Advanced Pharmacy Australia, a peak pharmacy body in Australia. Aged care pharmacists within the investigators’ professional networks will be invited to share recruitment materials with colleagues at RACHs who may be eligible to participate.

Residents or family members of residents who have an existing personal or professional relationship with the investigators will be excluded to avoid ethical and bias concerns regarding dependent relationships.

Participants will be provided with an honorarium for their participation in line with recommendations from the consumer advisory group and public guidance for participant honoraria, in the form of physical or electronic gift cards or cash payment.

### Output

The primary output of this project will be the co-design of a training program based upon the needs and preferences of stakeholders. This will include the overall modalities of training and assessment, activity guide, learning outcomes, and duration.

A secondary output will be an exploration of the needs and preferences of ACOPs when undertaking training for novel, professionally isolated roles.

### Framework and Timeline

This project will implement 2 layers of consumer and community consultation to achieve its objectives. A consumer advisory group will be consulted regularly through virtual meetings every month to guide the project broadly, with more comprehensive stakeholder engagement in small-group workshops.

**Figure 1.**
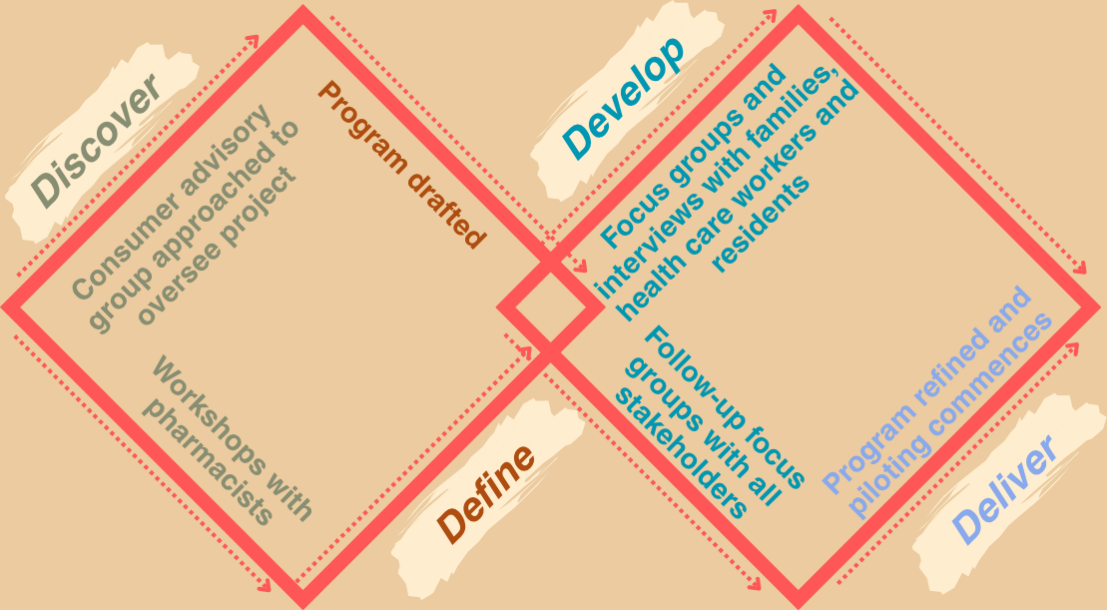
Study assets mapped to the Double Diamond process.

The co-design project will be guided by the Double Diamond process, a framework for innovative design that has been used in previous aged care innovations [47,48]. It involves 4 stages of alternating divergence (deep and exploratory thinking) and convergence (focused actions). The 4 stages and a summary of study-specific components of each stage are shown in Figure 1 and Table 1.

**Table 1.** Implementation of the Double Diamond design model.

Double Diamond stage	Summary of purpose	Summary of process
Discover	Explore the training and development needs and preferences of aged care on-site pharmacists and generate preliminary ideas for a training program. Explore perceptions of community members on the roles of pharmacists in residential aged care homes and how a training program for pharmacists could support these roles.	The CAG^a^ is engaged and informed on the goals of the project. The CAG is consulted on preliminary workshop plans, particularly regarding non–health professional participants (including logistics such as timing and location, feedback on appropriateness of activities, facilitator and discussion guide, and more). Early exploratory workshops with pharmacists are undertaken to identify the overall needs of on-site pharmacists and inform future workshops. A facilitator guide is attached in Multimedia Appendix 1. Exploratory questions were designed with the assistance of a specialist design department Match Studio [49]. Emblematic prompt questions are featured below, including the Mentimeter functionalities if used. All functionalities such as scale rating or word cloud activities are designed to be followed up with open discussion and further questions from the facilitator. Inductive, reflexive thematic analysis will be undertaken with focus group data to identify themes as well as discrete preferences of pharmacists. Focus group prompting questions: *How prepared were you when beginning work as an onsite pharmacist?* [Scale rating question] *Do you currently feel well-supported as an onsite pharmacist?* [Scale rating question] *Do you feel confident in your knowledge and skills as an onsite pharmacist?* [Scale rating question] *What words describe the ideal aged care pharmacist?* [Word cloud activity] *What would the perfect onsite pharmacist role look like?* [Open discussion] *What clinical skills or knowledge are most important for onsite pharmacists?* [Word cloud activity]
Define	Identify existing training program frameworks suitable for needs identified and perceptions of stakeholders and collaborate with organizations to refine existing frameworks.	A draft program will be developed in consultation with Advanced Pharmacy Australia and the research project team. Findings from concurrent studies, described above, will be integrated as available throughout the define and develop process. The CAG will be consulted for feedback and input on the developed draft, with recommendations implemented for presentation in follow-up workshops.
Develop	Explore perceptions of consumers and community members on the developed program and explore any alternative or divergent beliefs or recommendations.	Workshops with residents, family members of residents, and aged care staff will be undertaken. Questions will be include those exploring pharmacist roles and required skills and knowledge, in addition to questions expanding on findings from earlier pharmacist workshops. These will particularly explore potential barriers and facilitators for implementation. The structure of the training program and proposed training materials will be further refined using insights gathered from the Discover and Develop workshops, in consultation with Advanced Pharmacy Australia. The program will then be presented to pharmacist participants, residents and family members, and other health care staff to obtain feedback and recommendations for further refinement. Further details on implementation and evaluation methods will be sought. Depending on the level of agreement during focus groups and the extent of refinements suggested, further follow-up workshops will be undertaken if significant changes are made.
Deliver	Pilot and evaluate program, with modifications ongoing following feedback and consultation with participants.	The training program draft will be implemented and evaluated. Evaluation will involve feedback from participants in the pilot phase. The precise evaluation methodology will be determined in consultation with stakeholders throughout the workshops as part of the co-design process.

a CAG: consumer advisory group.

Overall design and content will be confirmed through interaction and dialogue among the research team, the pilot participants and the consumer advisory group. Differences in opinion will be resolved with collaborative discussions to reach mutually agreed decisions.

### Consumer Advisory Group

A consumer advisory group (CAG) has been organized via the Western Australia Health Translation Network as an integral component of the research team. This group brings lived experience expertise to collaborate with the research team for both high-level overarching guidance and specific insights. The CAG will be used for initial idea-generating and guidance for the project’s suitability from a resident and family perspective. The group comprises persons with personal and professional experience in navigating the aged care system, including with culturally and linguistically diverse persons. Both exploratory and tailored questions will be brought to this group to inform the training and development program broadly, as well as to adapt the workshops to optimize information-gathering and appropriateness for participants. This group will be consulted on a monthly basis across the planning, design, implementation, and evaluation stages.

The primary objective of the CAG is 2-fold. It will collaboratively guide the overarching training program development to ensure it meets the needs of stakeholders and support the design and development across workshop stages. Input into workshops will cover aspects such as development of the facilitation guide and questions, remuneration and acknowledgment of participants, durations of workshops, and ensuring the comfort and safety of workshop participants.

Members of the CAG will be remunerated for their time and participation. They will also be acknowledged as coauthors on any publications for which they meet authorship criteria. This aligns with recommendations on acknowledgment for consumer and community participation [50].

### Workshops

Interactive workshops will be used for more focused exploration and engagement. The workshops aim to explore and identify the preferences and needs of current and future ACOPs, health care staff, residents, and families or informal caregivers, as they pertain to the training needs of pharmacists. Preliminary findings from the 2023 national stakeholder meeting will inform some directed questions within the facilitator guide [1]. However, given the novelty of the ACOP intervention, questions will largely remain exploratory in the early stages, with subsequent workshops building on iterative findings. These broad and exploratory concepts will focus on concepts such as “the ideal role of a pharmacist in aged care” and “what people new to aged care practice need to know.”

Workshops will use a focus group–style structure with a facilitator guide including semistructured prompt questions, guardrails, and clear intentionality. A co-design expert (AD) will be consulted in the development of the workshop guides to ensure opportunities for divergent thinking and creativity are present. One author (TM) will facilitate all workshops, with the initial 2 to 3 additionally overseen by either investigators JJ or AD.

A strategic combination of virtual and in-person workshops will be used to suit the diverse spatiotemporal needs and availability of the cohorts in this project [51]. Given the geographic distribution and temporal availability of professional participants, virtual workshops were selected as a pragmatic choice. For persons residing in aged care, in-person workshops were deemed most appropriate to optimize participant comfort and involvement.

Virtual workshops will be supported through the use of Mentimeter software [52]. This software facilitates the use of various interactive activities to foster creativity and participation, including polls, open-ended questions, and anonymous response options [53]. Workshops conducted with residents will be conducted in person in a private room within their RACH to optimize comfort and accessibility. This decision was made following preliminary consultation with the CAG, as well as the research team, who have experience in conducting co-design research in residential aged care.

Three development rounds of workshops will be undertaken with the various stakeholder groups, followed by a piloting and evaluation phase of the developed program characterized by consultation with participants in the program for in-depth participatory research. Incrementally developed ideas and prototypes will be presented based on previous rounds of workshops. The various stages of workshops and CAG consultation are detailed in Table 1, in line with the Double Diamond framework.

Feedback will be obtained following completion of workshops in the form of brief questionnaires using Likert scales, to be developed with AD (a co-design expert). These questionnaires will focus on participants’ overall perceptions of the completed program, as well as their perceived level of involvement in the consultation process. This will be reported in final publications for transparency.

### Pilot Phase

Following development of the program after initial stakeholder consultation, recruitment will be undertaken for pharmacists to participate in a longitudinal education program, redesigned as required throughout the process in response to ad hoc feedback from participants. A separate evaluation project using scientific realism to explore what participants perceive to be the outcomes and how these outcomes have occurred will be undertaken.

### Data Collection

All workshops will be audio- and video-recorded, with data stored on a password-protected Adelaide University SharePoint folder. Transcripts will be automatically transcribed using Zoom software (Zoom Video Communications, Inc.) and subsequently checked and updated for accuracy by the facilitator.

Online workshops using Mentimeter will use surveys and word clouds, the data from which will be recorded and stored electronically. Physical data collected during in-person workshops, such as written, drawn, or other physical forms of participation, will be electronically scanned and stored with other electronic data in password-protected folders.

Supplementary qualitative interviews may be undertaken, with participants purposively sampled if deemed necessary for the project to address the co-design project objectives.

### Data Analysis

Recorded data, including physical and audio recordings from in-person workshops and one-on-one interviews, will be imported into NVivo software (Lumivero) for analysis of both trends and divergent perspectives, both of which are valuable in co-design research. The primary researcher, who is an on-site pharmacist himself, will undertake the thematic analysis in accordance with guidance described by Braun and Clarke [54]. This training program will be refined as needed following each stakeholder group consultation, in anticipation of upcoming workshops.

**Figure 2.**
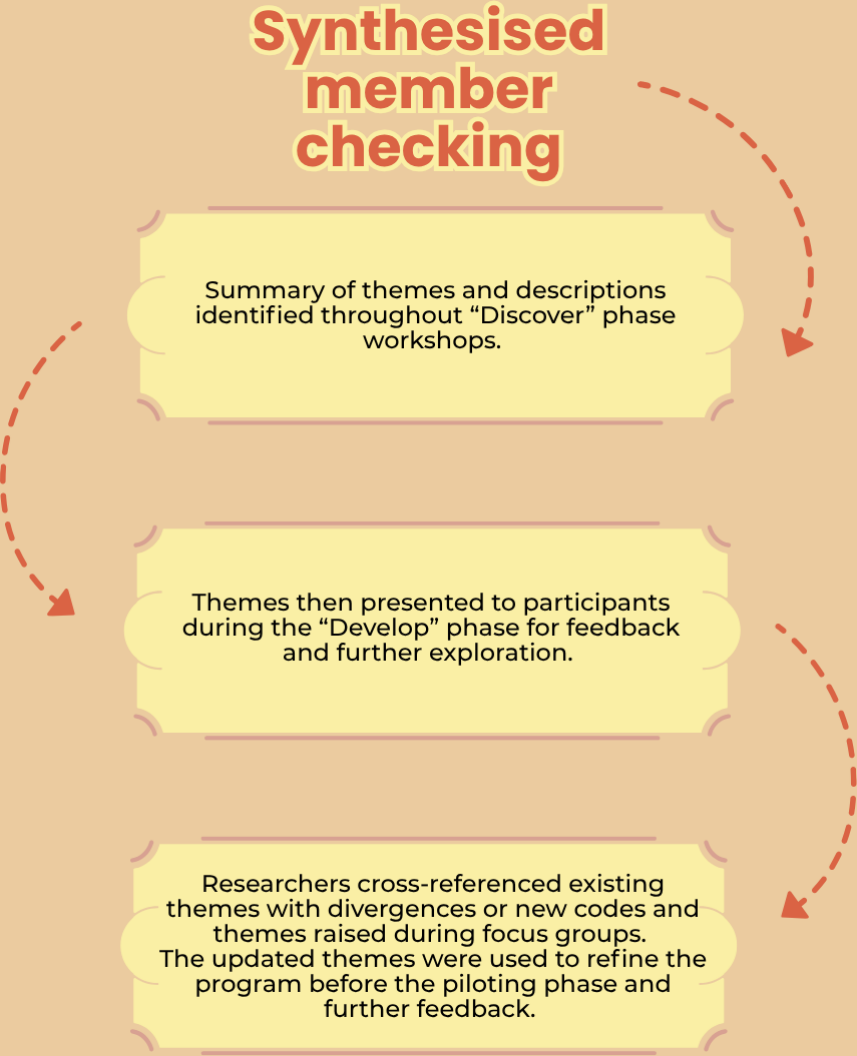
Synthesized member checking, adapted from the study by Birt et al [58].

The thematic analysis process will involve deep immersion in the data through participation as a facilitator as well as transcription checking and rereading. Initial line-by-line coding will then be undertaken with individual transcripts following each workshop. Codes will then be grouped into descriptive themes, remaining close to the data in line with qualitative descriptive methodology [55-57]. Participant validation will be undertaken by discussing initial codes and preliminary descriptive themes from the “Discover” workshops with other researchers and the CAG to identify overarching analytic themes, update facilitator guides for future workshops, and develop preliminary models for a training and development program. This will be followed by using a modified form of Synthesized Member Checking (SMC) as described by Birt et al [58]. In this phase, workshops throughout the “Develop” stage will seek to confirm themes from preliminary workshops following operationalization of such themes, as well as obtain feedback on the draft program. The modified form of SMC is shown in Figure 2.

Various strategies will be used to maintain rigor and trustworthiness. The primary researcher will leverage their professional insights and remain authentically connected with the data. They will ensure integrity, reflexivity, and criticality by acknowledging their positionality, maintaining an audit trail and reflexive journal, and ensuring a transparent decision-making process discussed with the wider research team [59,60]. Authenticity, credibility, and confirmability will be maintained through verbatim transcription, data-driven content analysis, the categorization of codes, and the use of exemplars.

### Decision-Making and Power Dynamics

Co-design is often characterized by a shared decision-making process where the relative expertise of the stakeholders and researchers is leveraged in a collaborative decision-making approach. Deep reflection is required to understand the extent to which this truly empowers end users, and these processes are not as straightforward as consensus decision-making given the potential for “legitimating and deepening” paternalistic and hierarchical power structures [61,62]. This challenge is particularly relevant in novel and complex interventions wherein stakeholders’ lived experience expertise may not provide the requisite context to make informed decisions, or true decision-making power sits outside the stakeholders’ locus of control. As outlined in the Methods section above, in this project we will focus primarily on enabling participants to contribute their knowledge and expertise to the process [43].

The ACOP role itself, as well as an education program tailored to this role, are both novel in the Australian setting. Therefore, to balance research requirements while addressing potential or actual power dynamic imbalances, a pragmatic “good enough to try, safe enough to fail” consent-based approach will be undertaken [63,64]. Early workshops with pharmacists and other stakeholders will be exploratory in nature. Participants in the pilot phase will be engaged in the penultimate phase of the process using the consent-based approach before formal implementation. An iterative feedback process will then be inbuilt into the final program to ensure a feedback loop continues with the end users. This feedback process will be administered by Advanced Pharmacy Australia when the program is made available through its platform.

Reflexivity will be maintained by the primary investigator in the form of a reflexive diary, as well as through the maintenance of an audit trail. This will be used not only for the purposes of rigor in data analysis but across the participatory process to ensure transparency in the decision-making process and to acknowledge potential or actual power dynamics that may emerge.

### Ethical Considerations

Research ethics approval has been obtained from the University of South Australia Human Research Ethics Committee (reference 206543). Consent for workshop participation will be sought if an individual is willing to participate and has capacity. All data will be deidentified prior to analysis. Participants will receive an honorarium for participation in workshops and/or interviews.

## Results

Funding for this project through the Medicines Research Future Fund was granted on March 27, 2023. Recruitment for preliminary phases began in January 2025, and data collection began in March 2025. Data collection is expected to end in October 2026. Ethics approval for this study was granted on December 19, 2024, by the University of South Australia Human Research Ethics Committee. Recruitment has exceeded the initial estimates of minimum participant involvement. Study findings are expected to be available in early 2027.

## Discussion

### Anticipated Findings

This project will use focus group–style workshops and oversight from a CAG to guide the development of the first training program available for pharmacists working in ACOP roles in Australia. It has been previously identified that pharmacists working in aged care roles in Australia, and indeed internationally, require ongoing, tailored education opportunities. Such programs should be able to develop clinical and nonclinical skills, assist in integrating into multidisciplinary health care teams, and provide pathways to professional recognition [17,18,65-67]. It is expected that the authentic partnership with end users and other stakeholders will assist in designing, developing, and implementing a novel program that suits the varied needs of all stakeholders.

### Conclusions

The final program will be the first co-designed workplace-based training program available for pharmacists working in the novel aged care on-site model. It is expected to strengthen pharmacists’ clinical decision-making skills, leadership, communication skills, and sphere of influence. By improving these capacities and capabilities, the program has the potential to enhance medication safety and optimize care quality within RACHs.

More broadly, this protocol demonstrates a scalable approach to co-designing workforce development interventions in complex care environments, offering a model that may be adapted to other novel health care roles.

## Supplementary material

10.2196/95889 Multimedia Appendix 1 Feedback questionnaires for participants of workshops.

## Abbreviations:

ACOP: aged care on-site pharmacist
CAG: consumer advisory group
RACH: residential aged care home
SMC: Synthesized Member Checking
