# Supplementary material for: Identification of Training Needs and Development of a Work-Integrated Training Program for Aged Care On-Site Pharmacists: Protocol for a Co-Design Study
Source: JMIR Res Protoc. 2026 Jul 31;15:e95889. doi: 10.2196/95889 (PMC13427065; doi:10.2196/95889)
Supplement: Multimedia Appendix 1 [file resprot-v15-e95889-s001.docx]

**Feedback Questionnaires for Workshops/Focus groups:**

Please rank your level of agreement with the below items, from ‘completely disagre’ to ‘completely agree’:

1. I feel that I have been given the opportunity to voice my thoughts and beliefs.
2. I feel like my opinions have been heard in this process.
3. I feel that onsite pharmacists could benefit from this workplace training and development program.
4. I feel that this program draft requires minor revisions.
5. I feel that this program draft requires significant revisions.
